# Discordant Susceptibilities of Enterobacterales to Different Tetracycline Classes

**DOI:** 10.7759/cureus.74917

**Published:** 2024-12-01

**Authors:** Hannah Flores, Paul Luethy, James B Doub

**Affiliations:** 1 Infectious Diseases, University of Maryland Medical Center, Baltimore, USA; 2 Pathology and Laboratory Medicine, University of Maryland School of Medicine, Baltimore, USA; 3 Internal Medicine, University of Maryland Medical Center, Baltimore, USA

**Keywords:** antimicrobial resistance, enterobacterales, minocycline, omadacycline, tetracycline

## Abstract

When bacteria are tetracycline- or doxycycline-resistant, the ability of these bacteria to be susceptible to the other tetracyclines is not well defined. Consequently, gaining knowledge about the ability to infer Enterobacterales susceptibility to minocycline and third-generation tetracycline antibiotics from surrogates is vital. In this study, we show that tigecycline may be a reasonable surrogate from which clinicians can infer omadacycline and eravacycline susceptibilities, even in the presence of doxycycline and tetracycline resistance. Yet, minocycline susceptibility cannot be inferred from third-generation tetracyclines used as surrogates when Enterobacterales is resistant to doxycycline and tetracycline.

## Introduction

Tetracyclines have a broad spectrum of activity against Gram-positive and Gram-negative bacteria, and they have favorable pharmacokinetic/pharmacodynamic (PK/PD) properties, making them advantageous antibiotics for a variety of infectious conditions [1]. Furthermore, the rise of multidrug-resistant organisms (MDROs) has prompted the recycling of historical tetracyclines, such as minocycline, and the development of new third-generation tetracyclines, which include eravacycline, tigecycline, and omadacycline. These newer tetracyclines have overcome some of the mechanisms of acquired tetracycline resistance, making them beneficial agents in the treatment of MDROs [2].

Although the second-generation tetracyclines, doxycycline and minocycline, vary in their serum half-life and lipid solubility compared to first-generation tetracyclines, in vitro first-generation tetracycline susceptibility has been used as a surrogate for doxycycline and minocycline susceptibilities [1,3,4]. Yet, when bacteria are tetracycline- or doxycycline-resistant, the ability of these bacteria to be susceptible to the other tetracyclines is not well defined. Small studies have shown the retention of minocycline susceptibility when Gram-positive organisms were tetracycline- and doxycycline-resistant, but few have investigated this in relation to Gram-negative infections [4,5]. Consequently, the aim of this study was to assess the ability of Enterobacterales to retain minocycline, eravacycline, and omadacycline susceptibilities despite resistance to tetracycline and doxycycline. 

## Materials and methods

This study was approved by the University of Maryland Internal Review Board (HP-00108256). Previously well-preserved (-80ºC glycerol stock) *Escherichia coli *(n=10), *Klebsiella pneumoniae* (n=10), and *Enterobacter cloacae* (n=10) clinical isolates from January 2021 to January 2024, which were resistant to tetracycline and doxycycline but sensitive to tigecycline based on the Vitek II Gram-negative bacilli (GN74) susceptibility card (Biomerieux, Durham, NC), were used for this study. These isolates were selected based on the availability of preserved isolates during the temporal time period. 

To evaluate various tetracycline susceptibilities, individual clinical isolates were cultured in tryptic soy broth from frozen glycerol stocks. The isolates were subcultured and then inoculated into tryptic soy broth and grown overnight at 37°C. A small inoculum of overnight growth was then placed into saline to create a 0.5 McFarland solution, and cotton swabs were used to create a lawn of bacterial growth on Mueller Hinton agar plates. Kirby-Bauer discs of doxycycline (30 µg), eravacycline (20 µg), tigecycline (15 µg), and omadacycline (30 µg) (Hardy Diagnostics, Santa Maria, CA) were then placed on the freshly inoculated plates. These discs were chosen based on the availability of tetracycline discs and on the potential tetracyclines that could have been clinically used to treat these infections. Each isolate was tested against the antimicrobial agents in triplicate, and the tests were repeated. The European Committee on Antimicrobial Susceptibility Testing (EUCAST) clinical breakpoints were utilized, with the zone of growth inhibition diameter breakpoints of ≥17 mm considered sensitive and <17 mm considered resistant. 

Moreover, susceptibility to various tetracyclines was also assessed using microbroth dilution assays, in which freshly prepared Mueller Hinton broth, made on the day of experimentation, was used. Falcon 96 microwell plates (Corning, Corning, NY) were utilized to conduct the experiment, in which serially diluted concentrations of tigecycline, eravacycline, and omadacycline (MedChemExpress, Monmouth Junction, NJ) and tetracycline, minocycline, and doxycycline (Sigma Aldrich, Burlington, MA) were placed into wells in the microwell plates. The choice of tetracyclines used was based on their potential clinical use to treat these infections. Again, 0.5 McFarland solution of overnight growth of the various individual bacteria was then placed in the microwells as indicated. Optical density (600 nm) was measured at 18 hours using SpectraMax iD5 (Molecular Devices, Sunnyvale, CA). Minimal inhibitory concentrations (MICs) were then compared to positive and negative controls. The Minocycline Epsilometer test (E-test) (Biomerieux, Marcy-l'Étoile, France) was also utilized and conducted in a similar manner to the Kirby-Bauer disc diffusion assays on Mueller-Hinton agar plates, given minocycline Kirby-Bauer discs are not readily available. EUCAST clinical breakpoints were utilized, with an MIC of ≤0.5 mg/L considered sensitive and >0.5 mg/L considered resistant. If there were differences in MIC values, the higher values were documented. 

## Results

**Figure 1:**
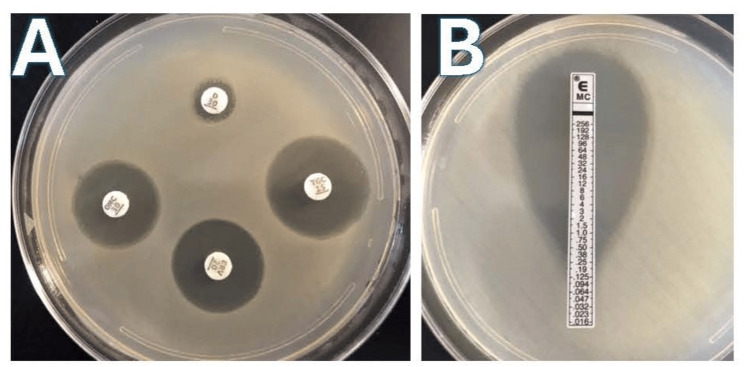
Images of Kirby-Bauer and E-test results. (A) Kirby-Bauer results for an individual isolate, in which zones of inhibition >20 mm were observed for tigecycline, omadacycline, and eravacycline and zones of growth inhibition <10 mm for doxycycline. (B) Epsilometer test for the same isolate, showing MIC of 0.25 mg/L. MIC: minimal inhibitory concentration; E-test: Epsilometer test.

All bacteria evaluated were resistant to both tetracycline and doxycycline based on Kirby-Bauer assessments and microbroth dilution assays, with zones of growth inhibition <10 mm and MIC over 4 mg/L (Figure 1a, Table 1). There was no difference in the MIC when comparing tetracycline to doxycycline for any of the clinical isolates. Interestingly, all 30 bacteria evaluated were susceptible to eravacycline, omadacycline, and eravacycline. This was observed with large zones of growth inhibition (>20 mm) with Kirby Bauer assays (Figure 1a) for all clinical isolates. When evaluated with microbroth dilution assays, these antibiotics also had low MICs (<0.25 mg/L) for all the clinical isolates evaluated. Moreover, the categorical outcomes of the Kirby-Bauer evaluation correlated with the categorical outcomes of microbroth dilution assay for each clinical isolate. 

Interestingly, when minocycline susceptibility was evaluated for the clinical isolates, there was discordance in activity. Seventy percent of the *E. coli *and *E. cloacae* clinical isolates were non-susceptible to minocycline, as seen with microbroth dilution and E-test (Figure 1b). Similarly, 60% of the *K. pneumoniae *clinical isolates were non-susceptible to minocycline. The clinical isolates that were minocycline-susceptible had low MICs, similar to those seen with eravacycline, omadacycline, and tigecycline, respectively, on both E-test (Figure 1b) and microbroth dilution (Table 1). 

**Table 1:** Microbroth dilution, Kirby-Bauer, and E-test susceptibility results of various clinical isolates to tetracyclines. Based on European Committee on Antimicrobial Susceptibility Testing guidelines, zones of growth inhibition diameter breakpoints of ≥17 mm were considered sensitive and <17 mm were considered resistant as well as MICs of ≤0.5 mg/L were considered sensitive and >0.5 mg/L were considered resistant. E-test: Epsilometer test; MICs: minimal inhibitory concentrations.

Organism (n)	Doxycycline-susceptible isolates n (%)	Minocycline-susceptible isolates n (%)	Tigecycline-susceptible isolates n (%)	Eravacycline-susceptible isolates n (%)		Omadacycline-susceptible isolates n (%)
*E. coli* (10)	0 (0)	3 (30)	10 (100)	10 (100)	10 (100)	
*E. cloacae* (10)	0 (0)	3 (30)	10 (100)	10 (100)	10 (100)	
*K. pneumoniae* (10)	0 (0)	4 (40)	10 (100)	10 (100)	10 (100)	

## Discussion

Tetracycline resistance is primarily acquired through tet genes, which are involved in antibiotic efflux, ribosomal protection, and enzymatic inactivation of tetracyclines [6]. Tetracycline-specific efflux pumps are the most common members of the major facilitator superfamily (MFS) of transporters and are composed of two groups. Group 1 includes *TetA* and *TetB*, and Group 2 includes *TetK *and *TetL*. *TetA* and* TetB* are the most common efflux pumps found in Gram-negative clinical isolates, while *TetK* and *TetL* are the most common in Gram-positive isolates [6]. Other important resistance mechanisms involve tetracycline ribosomal protection proteins (RPPs). These genes, transmitted on mobile genetic elements, are found in Gram-positive and Gram-negative organisms. The best-characterized RPPs are *TetO* and *TetM*, which catalyze the GTP-dependent release of tetracycline from the ribosome [6]. This mechanism confers resistance to tetracycline, minocycline, and doxycycline, but tetracyclines containing bulkier side chains at the C9 position, such as tigecycline, eravacycline, and omadacycline, retain activity despite the presence of these genes [6]. Finally, tetracycline-modifying enzymes are plasmid-conferred genes encoded by *TetX *and inactivate tetracyclines by the addition of a hydroxyl group between the C and B rings of the tetracycline core [6].

Knowledge of the different *tet* genes is important for clinicians to be cognizant of, but at present, determining the presence or absence of these genes in clinical bacterial isolates is not readily conducted. Consequently, gaining knowledge about the ability to infer the susceptibility of minocycline and third-generation tetracycline antibiotics from surrogates is vital. The data presented here highlight the spectrum of Enterobacterales resistance that exists to tetracyclines. Based on our findings, we show that tigecycline may be a reasonable surrogate from which clinicians can infer omadacycline and eravacycline susceptibilities for Enterobacterales, even in the presence of doxycycline and tetracycline resistance. This is important because omadacycline resistance testing is not readily available. However, given this was a pilot study, larger studies are needed to support this claim. 

While this study found that all the clinical isolates were susceptible to tigecycline, eravacycline, and omadacycline, the same was not true for minocycline. Higher rates of minocycline resistance were seen in our samples despite tigecycline susceptibility. Discordant minocycline susceptibility was previously demonstrated in Gram-positive peri-prosthetic joint infection clinical isolates, specifically in vancomycin-resistant *Enterococcus faecium* and *Enterococcus faecalis* [5]. Similarly, the variability in minocycline resistance for Enterobacterales is likely due to the acquisition of RPPs via genes on mobile genetic elements and differences in efflux pump specificity. Thus, it is important for clinicians to realize that minocycline susceptibility testing for individual isolates is vital, as minocycline susceptibility cannot be inferred from third-generation tetracyclines used as surrogates when Enterobacterales are resistant to doxycycline and tetracycline. 

This is a novel finding, and this has not been thoroughly evaluated beyond other studies evaluating single tetracycline agents [7]. However, we hypothesize that the difference in resistance could represent the presence of RPPs, given the bulkier side chains of third-generation tetracyclines allowed for continued susceptibility. Yet, the role of efflux pumps should also not be understated. While *TetA, TetB*, and *TetK* pumps recognize tetracycline, minocycline, and doxycycline, overexpression of *TetB* and *TetK* has no effect on tigecycline or eravacycline. Extrapolating from our other pilot study, since tetracycline resistance genes can be transmitted by mobile genetic elements, it is possible that the natural environment where bacteria reside plays an important role in acquiring certain tetracycline resistance genes [5]. This is because there are different evolutionary stressors leading to the transfer and acquisition of resistance genes in mammalian gut flora (where Enterobacterales and *Enterococcus* spp. reside) compared to mammalian skin and other locations [8]. This hypothesis is supported by the retention of minocycline susceptibility in numerous multidrug-resistant *Acinetobacter baumannii* [9,10]. In this study, we only evaluated phenotypic expression of susceptibilities to the different tetracycline classes and did not evaluate the presence of specific *tet* genes due to the lack of funding. Therefore, the presence of specific antibiotic resistance genes and their role in resistance in this study is hypothetical but should be further evaluated with adequate funding. 

Although the findings here are clinically relevant and can help guide clinicians, there are some limitations to our study. First, the sample size of clinical isolates was small (n=30), so the findings seen here will need to be evaluated in larger studies. Correlated with this is the fact that this study only evaluated three species of Enterobacterales, as these were the most common pathogens at our institution to cause clinical infections with some form of tetracycline resistance. Yet, larger follow-up studies should focus on other Enterobacterales. However, these larger studies will likely need to be multicenter collaborations to obtain an adequate number of samples in a temporally feasible timeframe. Lastly, we inferred minocycline, omadacycline, and eravacycline susceptibilities based on EUCAST breakpoints for other similar tetracyclines. While this is likely clinically applicable, there is a lack of data to support this inference, and therefore, follow-up studies are needed to support these findings.

## Conclusions

In conclusion, inferring resistance patterns among the different classes of tetracyclines when isolates are resistant to doxycycline is challenging due to variable mechanisms of resistance. However, our data suggest that it may be reasonable to infer the susceptibility of eravacycline and omadacycline based on tigecycline susceptibility in Enterobacteriaceae isolates. However, minocycline susceptibility should not be inferred from third-generation tetracycline susceptibilities when clinical isolates are resistant to doxycycline. Rather, clinical isolates require independent testing with E-tests or microbroth dilutions to determine an individual isolate’s minocycline susceptibility. Overall, this study has important clinical implications, but larger follow-up studies, especially with different infectious conditions, are needed to support the findings to allow for absolute clinical applicability. 
